# Supplementary material for: A Community-Engaged Approach to Community Health Needs and Assets Assessment for Public Health Research
Source: Int J Environ Res Public Health. 2025 Jun 27;22(7):1030. doi: 10.3390/ijerph22071030 (PMC12294907; doi:10.3390/ijerph22071030)
Supplement: Supplementary file 1 [file ijerph-22-01030-s001.zip › Figure S1.pdf]

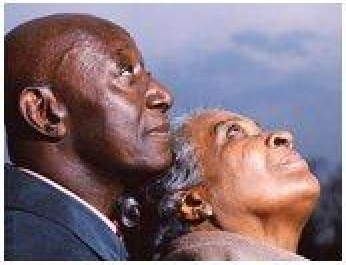
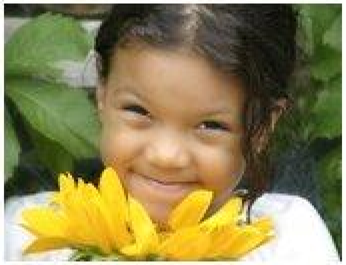
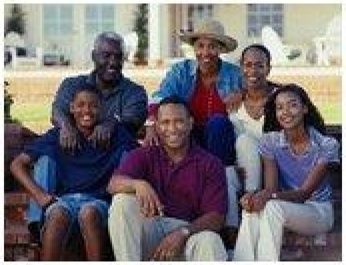

English

Introduction

2022 MSM PRC Community Health Needs & Assets Assessment Survey

The Morehouse School of Medicine Prevention Research Center is doing a Community Health Needs and Assets Assessment. As a neighborhood resident your views about the health concerns and resources in your community is important to us.

What you share will help us with health promotion programs and prevention education services that will benefit you and your community. Please take fifteen minutes of your time right now to complete this survey. **There are no right or wrong answers** and what you share will not be used to identify you. Please tell us what you think.

Before we begin – have you done this survey already this year?

- ◯ Yes
- ◯ No
- ◯ Not Sure

Before you proceed to the survey, please complete the captcha below.

☐ I'm not a robot

reCAPTCHA
Privacy - Terms

Bot Detection

Wilder Penfield stimulated the brains of patients undergoing brain surgery for which neurological disorder?

- Alzheimer's
- Epilepsy
- Multiple Sclerosis
- Depression

Background Information

Do you live in Fulton County?

- Yes
- No

Which community do you live in?

- Adair Park
- Amal Heights
- Ashview Heights
- Atlanta University Center
- Betmar La Villa
- Blair Villa / Poole Creek
- Browns Mill Park
- Capitol Gateway
- Capitol View
- Capitol View Manor
- Chosewood Park
- Glenrose Heights
- Hammond Park

- ◯ Harris Chiles
- ◯ High Point
- ◯ Joyland
- ◯ Just Us
- ◯ Lakewood
- ◯ Lakewood Heights
- ◯ Leila Valley
- ◯ Mechanicsville
- ◯ Norwood Manor
- ◯ Orchard Knob
- ◯ Peoplestown
- ◯ Perkerson
- ◯ Pittsburgh
- ◯ Polar Rock
- ◯ Rebel Valley Forest
- ◯ Rosedale Heights
- ◯ South Atlanta
- ◯ South River Gardens
- ◯ Summerhill
- ◯ Sylvan Hills
- ◯ The Villages at Carver
- ◯ The Villages at Castleberry Hill
- ◯ Thomasville Heights
- ◯ West End
- ◯ Westview
- ◯ Other (Please Specify)
- ◯ Don't Know

Which county do you live in?

- Appling
- Atkinson
- Bacon
- Baldwin
- Bleckley
- Calhoun
- Candler
- Charlton
- Clay
- Clayton
- Clinch
- Cobb
- Coffee
- Colquitt
- Crisp
- Decatur
- DeKalb
- Dooley
- Dougherty
- Elbert
- Emanuel
- Evans
- Gilmer
- Gordon
- Grady
- Greene
- Gwinnett

- Johnson
- Laurens
- Macon
- Mitchell
- Montgomery
- Polk
- Seminole
- Stephens
- Stewart
- Sumter
- Thomas
- Tift
- Toombs
- Troup
- Turner
- Ware
- Washington
- Wayne
- Whitfield
- Other (Please Specify)
- Don't Know

If you live in a Neighborhood Planning Unit (NPU), which one do you live in?

- T
- V
- X
- Y

◯ Z

◯ Other (Please Specify)

◯ Don't Know

◯ I Do Not Live In An NPU

What is your Zip Code?

Health Concerns

WE WOULD LIKE TO KNOW MORE ABOUT HEALTH CONCERNS IN YOUR COMMUNITY

What are the top three health issues your community needs to know more about?

(Rank only three health issues by writing a “1” in the box next to the first priority, the second priority “2”, and the third priority “3”).

My **COMMUNITY** needs to know more about:

- Arthritis
- Asthma
- Autoimmune Diseases (e.g., Lupus, Rheumatoid arthritis, Multiple sclerosis, Type 1 Diabetes)
- Cancer
- COVID-19
- Diabetes
- Environmental Health (e.g., air pollution, landfills, litter)
- Epilepsy and seizure disorders
- Heart Disease
- High Blood Pressure

- [ ] HIV/AIDS
- [ ] Maternal Health
- [ ] Men's Health
- [ ] Mental Health
- [ ] Nutrition
- [ ] Obesity
- [ ] Physical Disability
- [ ] Second-or Third-hand smoke
- [ ] Sexually Transmitted Diseases or Infections (e.g., Chlamydia, Herpes, Gonorrhea, Syphilis)
- [ ] Stroke
- [ ] Substance Abuse
- [ ] Teen Pregnancy
- [ ] Violence Prevention
- [ ] Women's Health
- [ ] Other (Please List) ______

For each of your top three choices, please tell us why the health issues you chose are important.

______

What do you think are the causes of the health issues/concerns you identified?

______

What do you think should be done to solve these health issues/concerns?

______

Please rank the top three health issues you would like to learn more about for yourself.

(Rank only three health issues by writing a “1” in the box next to the first priority, the second priority “2”, and the third priority “3”).

For my **OWN** health, I want to learn more about:

- [ ] Arthritis
- [ ] Asthma
- [ ] Autoimmune Diseases (e.g., Lupus, Rheumatoid arthritis, Multiple sclerosis, Type 1 Diabetes)
- [ ] Cancer
- [ ] COVID-19
- [ ] Diabetes
- [ ] Environmental Health (e.g., air pollution, landfills, litter)
- [ ] Epilepsy and seizure disorders
- [ ] Heart Disease
- [ ] High Blood Pressure
- [ ] HIV/AIDS
- [ ] Maternal Health
- [ ] Men's Health
- [ ] Mental Health
- [ ] Nutrition
- [ ] Obesity
- [ ] Physical Disability
- [ ] Second-or Third-hand smoke
- [ ] Sexually Transmitted Diseases or Infections (e.g., Chlamydia, Herpes, Gonorrhea, Syphilis)
- [ ] Stroke
- [ ] Substance Abuse
- [ ] Teen Pregnancy
- [ ] Violence Prevention
- [ ] Women's Health

Other (Please List)

For each of your top three choices, please tell us why the health issues you chose are important for you.

What are the top three policy, system, or environmental issues that need to be addressed in your community to improve health?

(Rank only three issues by writing a “1” in the box next to the first priority, the second priority “2”, and the third priority “3”).

For my **COMMUNITY**, I want the following to be addressed:

- Access to Health Insurance
- Access to Healthy Foods
- Access to the Internet/Technology
- Access to Physical Activity
- Access to Quality Healthcare Services
- Child Care
- Employment
- Health Education
- Housing
- Mental Health Treatment
- Poverty
- Public Education System
- Racism
- Sexism
- Substance Abuse
- Transportation
- Youth Employment

Youth Engagement (e.g., Recreation Centers)

Other (Please List)

For each of your top three choices, please tell us why the policy, system or environmental issues you chose are important.

Health Programs & Services

NOW, WE WOULD LIKE TO KNOW WHAT HEALTH PROGRAMS AND SERVICES ARE AVAILABLE TO YOU

Are there any health programs in your community (e.g., walking clubs, weight loss programs)?

- Yes (Please Specify)
- No
- Don't Know

Have you attended any health programs in your community?

- Yes (Please Specify)
- No

The Morehouse School of Medicine Prevention Research Center has a number of community health programs for community members. We would like your input on how to let people know about these programs.

Please rank your top three choices (Write "1" next to the choice that is the best, “2” the second best, and “3” the third best place).

Where do you think the top three places are to invite community members to hear about these programs?

Churches

- [ ] Community Events
- [ ] Daycare Centers
- [ ] Health Clinics
- [ ] Neighborhood Buisnesses
- [ ] Neighborhood Centers (Please specify name) ________
- [ ] Neighborhood Meetings
- [ ] Public Assistance Office
- [ ] Rentals Offices
- [ ] School Meetings
- [ ] Social Media (e.g. Facebook, Instagram, Twitter)
- [ ] Other (Please List) ________

Do you have health insurance?

- ○ Yes
- ○ No

If so, what? (Check all that apply)

	Yes	No
Insurance through a current or former employer or union	○	○
Insurance purchased directly from an insurance company	○	○
Medicare, for people 65 and older, or people with certain disabilities	○	○
Medicaid, Medical Assistance, or any kind of government-assistance plan for those with low incomes or a disability	○	○
TRICARE or other military health care	○	○
VA (including those who have ever used or enrolled for VA health care)	○	○
Indian Health Service	○	○
Grady Health Card	○	○
Any other type of health insurance or health coverage plan not listed (Please List) ________	○	○

If other, is your health insurance public or private?

- Public
- Private

Do you have supplemental insurance (i.e., additional insurance that pays directly to the insured such as AFLAC)?

- Yes
- No

A primary care doctor is your main doctor who you see regularly and helps manage your overall health that is not urgent care or the emergency room.

Do you have a primary care doctor?

- Yes
- No

How often do you see your primary care doctor?

- Annually (once a year)
- Monthly
- Quarterly (a few times a year)
- Weekly
- Other (Please specify):

Do you participate in any of the activities to promote good health? (Check all that apply)

	Yes	No
COVID testing	○	○
Eat healthy foods	○	○
Exercise daily on a regular basis	○	○

	Yes	No
Receive recommended screenings (e.g., cervical cancer screening, colonoscopy, mammogram))	○	○
See the dentist for routine dental exams	○	○
See the doctor for annual physical exams	○	○
Take a daily multivitamin or mineral supplement	○	○
Take children to receive scheduled shots (immunization)	○	○
Vaccinations (annual flu shot, COVID vaccination, Shingles vaccination)	○	○
Other (Please list)	○	○

Do you mostly seek health care in an emergency room?

- ○ Yes
- ○ No

Where do you usually get health care? (Check all that apply)

	Yes	No
Grady Memorial Hospital	○	○
Good Samraritan	○	○
Lakewood Health Center (Lakewood Avenue)	○	○
South Fulton Medical Center (Carver High School Campus)	○	○
South Fulton Medical Center (Cleveland Avenue)	○	○
Southside Medical Center (Ridge Avenue)	○	○
Atlanta Medical Piedmont	○	○
Primary Care Physician (Please List Location)	○	○
Urgent Care (Please List Location)	○	○
Veterans Administration Hospital (VA)	○	○
Nowhere, Home Remedies Only	○	○
Walk In Clinics (i.e., CVS, Walgreens,)	○	○
Others (Please List)	○	○

What services/resources are available in your community? (Check all that apply)

	Yes	No
Community clinic	◯	◯
Counseling Services	◯	◯
Home health	◯	◯
Hospital	◯	◯
Private clinic	◯	◯
Urgent Care	◯	◯
YMCA (or other community clinic)	◯	◯
Other (Please List)	◯	◯

Health Service & Resource Information

NOW, WE WOULD LIKE TO KNOW THE BEST WAY TO SHARE HEALTH SERVICE AND RESOURCE INFORMATION WITH YOU

What are the three best ways to share health information with you?

Please rank your top three choices (Write "1" next to the choice that is the best, “2” the second best, and “3” the third best way).

- [ ] Attend Church Events
- [ ] Attend Community Events
- [ ] Email
- [ ] E-News Bulletin/E-Health Cards
- [ ] Facebook
- [ ] Flyers
- [ ] Gyms
- [ ] Health Clinics
- [ ] Health Fairs

- [ ] Instagram
- [ ] Internet
- [ ] Local Newspapers
- [ ] Mailers
- [ ] Neighborhood Meetings
- [ ] Phone
- [ ] Posters
- [ ] Radio Programs
- [ ] School Meetings
- [ ] Television Programs
- [ ] Text Messaging
- [ ] Tik Tok
- [ ] Twitter
- [ ] WhatsApp
- [ ] Word of Mouth
- [ ] YouTube
- [ ] Other (Please List) ________

Demographic Information

Please share any other ideas or comments about health concerns for you and your community.

____

NOW, WE WOULD LIKE TO KNOW ABOUT MORE ABOUT YOU

What is your age? (Check One)

- ○ 18-24 years

- 25-34 years
- 35-44 years
- 45-54 years
- 55-64 years
- 65 years or older

What is your gender?

- Male
- Female
- Transgender female or trans woman
- Transgender male or trans man
- Nonbinary, genderqueer, or genderfluid
- I would describe my gender as:
- Prefer not to answer

Are you Hispanic or Latino?

- Yes
- No

What do you consider your race to be? (Check all that apply)

	Yes	No
American Indian or Alaskan Native		
Asian		
Black/African American		
Native-Hawaiian or Other Pacific Islander		
White		

	Yes	No
Other (Please Specify)	◯	◯

Now thinking about your physical health, which includes physical illness and injury, for how many days during the past 30 days was your physical health not good?

- ◯ 0 days
- ◯ 1-7 days
- ◯ 8-14 days
- ◯ 15-21 days
- ◯ 22-29 days
- ◯ 30 days

Now thinking about your mental health, which includes stress, depression, and problems with emotions, for how many days during the past 30 days was your mental health not good?

- ◯ 0 days
- ◯ 1-7 days
- ◯ 8-14 days
- ◯ 15-21 days
- ◯ 22-29 days
- ◯ 30 days

Do you have a substance use issue?

- ◯ Yes
- ◯ No

How much do you trust each of these sources to provide correct information about your health? (Select one response for each row.)

	Not At All	A Little	A Great Deal	Don't Know
Your doctor or health care provider	◯	◯	◯	◯
Your faith leader (e.g., pastor, priest, etc.)	◯	◯	◯	◯
Your close friends and members of your family	◯	◯	◯	◯
People you go to work or class with or other people you know	◯	◯	◯	◯
News on the radio, TV, online, or in newspapers	◯	◯	◯	◯
Your contacts on social media	◯	◯	◯	◯
The U.S. government	◯	◯	◯	◯

What Neighborhood Planning Unit (NPU) do you work in?

- ◯ T
- ◯ V
- ◯ X
- ◯ Y
- ◯ Z
- ◯ I am not currently employed
- ◯ I do not work in any of the NPUs listed (Please Specify)
- ◯ Don't Know

What is your total family household income in a year?

- ◯ Under $10,000
- ◯ $10,001-$25,000
- ◯ $25,001-$40,000
- ◯ $40,001-$55,000
- ◯ $55.001-$75,000
- ◯ $75,001-$100,000

- Over $100,000
- I prefer not to disclose my income.

What is your marital status.

- Single, Never Married
- Informally Married or Living with a Permanent Partner
- Married
- Divorced
- Widowed
- I prefer not to disclose this information.

What is your sexual orientation.

- Bisexual
- Gay
- Heterosexual
- Lesbian
- Other (please specify)
- I prefer not to disclose this information.

Please tell us more about the children in your household (Check all that apply).

	Yes	No
Live with children under 6 years old	○	○
Live with children 6 to 17 years old	○	○
No children under 18 years old in the household.	○	○
I prefer not to disclose this information.	○	○

Please indicate your household size.

- 1-person-household
- 2-person-household
- 3-person-household
- 4-person-household
- 5-person-household
- 6-person-household
- 7-or-more-person household
- I prefer not to disclose this information.

Compensation

THANK YOU FOR TAKING TIME TO COMPLETE THIS SURVEY!
Please proceed to the next page to provide your information to receive your incentive. Incentives will be disseminated in order of completion.

First Name

Last Name

Email Address

City

County

State

Zip Code
