# Supplementary material for: A Community-Engaged Approach to Community Health Needs and Assets Assessment for Public Health Research
Source: Int J Environ Res Public Health. 2025 Jun 27;22(7):1030. doi: 10.3390/ijerph22071030 (PMC12294907; doi:10.3390/ijerph22071030)
Supplement: Supplementary file 1 [file ijerph-22-01030-s001.zip › Table S1.pdf]

Table S1: Health Concerns expressed by respondents, CHNAA Survey, 2022

Top 10 Community Health Concerns	
**Health Issue**	Frequency/ Percentage
1. **Diabetes**	183 (24%)
2. **COVID-19**	176 (23%)
3. **Mental Health**	154 (20%)
4. **High Blood Pressure**	151 (20%)
5. **Heart Disease**	128 (17%)
6. **Cancer**	126 (17%)
7. **Environmental Health**	112 (15%)
8. **Obesity**	95 (13%)
9. **Autoimmune Disease**	89 (12%)
10. **Substance Abuse**	75 (10%)
**10. HIV/ AIDS**	75 (10%)
**Top 10 Individual Health Concerns**	
1. **COVID-19**	140 (19%)
1. **Mental Health**	140 (19%)
2. **Diabetes**	137 (18%)
3. **High Blood Pressure**	135 (18%)
4. **Cancer**	126 (17%)
5. **Environmental Health**	124 (16%)
6. **Heart Disease**	116 (15%)
7. **Nutrition**	115 (15%)
8. **Women's Health**	111 (15%)
9. **Obesity**	100 (13%)
10. **Autoimmune Disease**	98 (13%)
**Top 5 Health-Related Policy, System, or Environmental Issues**	
1. **Access to Healthy Foods**	236 (31%)
2. **Access to Health Insurance**	218 (29%)
3. **Mental Health Treatment**	172 (23%)
4. **Access to Quality Healthcare Services**	150 (20%)
5. **Housing**	138 (18%)
